# Androgenic response of Triticum durum-Dasypyrum villosum amphidiploids and their parental forms

**DOI:** 10.18699/VJGB-22-17

**Published:** 2022-03

**Authors:** H. Stoyanov, I. Belchev

**Affiliations:** Dobrudzha Agricultural Institute, General Toshevo, Bulgaria; Dobrudzha Agricultural Institute, General Toshevo, Bulgaria

**Keywords:** anther culture, androgenic response, amphidiploid, Dasypyrum villosum, parental forms, пыльниковая культура, андрогенетическая реакция, амфидиплоид, Dasypyrum villosum, родительские формы

## Abstract

Wide hybridization in cereal crops is one of the most efficient tools for the enrichment of genetic variability and addressing a number of breeding problems related to resistance and tolerance to biotic and abiotic stresses. Therefore, a large number of amphidiploids between species possessing different morphological, genetic and physiological properties have been developed. One of the most valuable species with regard to the possibilities for introducing valuable traits and properties into wheat species is the wild Dasypyrum villosum. With the aim to study the androgenic response of the Triticum durum-D. villosum amphidiploids, two accessions and their parental forms – the durum wheat cultivars Gergana and Argonavt and a landrace of the D. villosum – were studied. The following parameters were determined: callus induction, plant regeneration, yield of albino and green regenerants. It was found that the callus induction of the two studied amphidiploids differed significantly from that of the parental forms (2.1–7.2 %), being significantly higher, 30.7 and 16.5 %, respectively. Regardless of the difference in callus induction, the amphidiploids did not significantly differ from the parental forms in their regeneration ability. The yield of albino plants exceeded the yield of green regenerants and followed the tendency observed in callus induction. Green plants were found only in the amphidiploid Gergana-D. villosum and in the parental form durum wheat Gergana. Plants were regenerated from the species D. villosum, although they were only albinos, showing its good responsiveness to anther culture. The established characteristics of the amphidiploids and their parental forms make their practical use highly valuable for the improvement of different types of cereal crops.

## Abstract (ru)

Отдаленная гибридизация злаков является одним из самых эффективных способов обогащения генетического разнообразия и решения множества селекционных задач в отношении устойчивости и толерантности к биотическому и абиотическому стрессу. Поэтому создано большое количество амфидиплоидов между отдельными видами, которые наделены разными морфологическими, генетическими и физиологическими характеристиками. Дикий вид Dasypyrum villosum – один из самых ценных видов с точки зрения возможности интродуцирования ценных качеств и свойств в разные сорта пшеницы. Для изучения реакции амфидиплоидов Triticum durum-D. villosum исследованы два образца, их родительские формы – сорта твердой пшеницы Гергана и Аргонавт, а также местная популяция вида D. villosum. Установлены следующие параметры: индукция каллусов, частота всех проростков к 100 эмбриоподобным структурам, частота альбиносных проростков и частота зеленых проростков. По полученным результатам выявлено, что индукция каллусов двух амфидиплоидов статистически достоверно отличается от родительских форм (2.1–7.2 %), будучи значительно выше, 30.7 и 16.5 % соответственно. Несмотря на разницу в индукции каллусов, амфидиплоиды практически не отличаются от родительских форм своей регенерирующей способностью. Частота альбиносных проростков значительно превышает частоту зеленых проростков, следуя тенденции, наблюдаемой в индукции каллусов. Зеленые проростки зарегистрированы только у амфидиплоида Гергана-D. villosum и у родительской формы твердой пшеницы Гергана. Растения вида D. villosum были регенерированы, и то, что были только альбиносные проростки, показывает хорошую отзывчивость вида на культивирование пыльников. Установленные характеристики испытанных амфидиплоидов и их родительских форм делают практическое использование этих амфидиплоидов особенно ценным для селекции различных видов злаков.

## Introduction

The development of highly productive varieties of cultivated plants, which at the same time are characterized with stable yields and are resistant to different biotic and abiotic stress factors, is a primary task in plant breeding (Chahal, Gosal, 2000). However, the increase of yield within the genome of a given species is not limitless (Grassini et al., 2013). In this respect, there are different approaches to enrich the genome of the cultivated plants – wide hybridization, genetic engineering, genome editing technologies, etc. (Chahal, Gosal, 2000; Liu et al., 2014; Okada et al., 2019; Li, 2020; Wang et al., 2020).

Although contemporary science has reached high levels of use of the latter two technologies, wide hybridization remains a main conventional tool for achieving high genetic variability. There is a large amount of research on amphidiploids de-veloped through wide hybridization among the cereal crops (Zhang et al., 2010; Ming et al., 2011; Babaiants et al., 2012; Stoyanov, 2013, 2014; Dai et al., 2015; Nemeth et al., 2015; Song et al., 2019; Klimushina et al., 2020; Zuo et al., 2020; Kiani et al., 2021). One of the most promising species for enrichment of the genome of common and durum wheat, however, is Dasypyrum villosum. This species has been described in detail with regard to the possibility of being used in the improvement work on the wheat species in the researches of A. Grądzielewska (2006a) and C. De Pace et al. (2011). In another research, A. Grądzielewska (2006b) described in detail a large number of studies on the production of hybrids, natural hybrids, substitution and addition lines with wheat and other species. There are a number of studies on the possibility of using the hybrids and amphidiploids of the wheat species with D. villosum in practical breeding (De Pace et al., 2001; Vaccino et al., 2010; De Pace et al., 2011; Zhang et al., 2015, 2016a, b, 2018; Ando et al., 2019). A. Stefani et al. (1987) reported rather detailed morphological characteristics of the amphidiploid Triticum durum-D. villosum.

Since plant breeding is a rather dynamic process, when developing lines from the cereal species, the biotechnological method of anther culture is often used to accelerate the breeding process (Belchev, 2003; Lantos, 2009). Different researchers report that the efficiency of the process and the production of a high number of green plants is related to the response to anther culture of the parental forms involved in the cross (Zamani et al., 2003; Dagüstü, 2008; Yildirim et al., 2008; El-Hennawy et al., 2011). In this respect, the developed amphidiploids, substitution and addition lines with D. villosum, are specific parental forms, the reaction to anther culture of which has not been studied up to now. The possibility to apply anther culture to amphidiploids in principle has been little investigated. The response to anther culture in the amphidiploids has been studied in the amphidiploid Aegilops variabilis-Secale cereale (Ponitka et al., 2002), and the authors determined 0.1–13.4 % of regenerants obtained from 100 androgenic embryoids. D. Plamenov et al. (2009) determined 1.9–3.2 % of green regenerants from 100 cultured anthers in the amphidiploid T. durum-T. monococcum ssp. aegilopoides. In tritordeum (Barcelo et al., 1994), it was also found out that anther culture is an efficient process. The results from these researches showed that different amphidiploids are able to give positive response to anther culture.

The aim of this study was to determine the reaction of the amphidiploid T. durum-D. villosum to anther cultivation in comparison to its parental forms.

## Materials and methods

Plant material. Two accessions of the amphidiploid T. durum- D. villosum (1dv (Gergana-D. villosum) and 2dv (Argonavt- D. villosum)), a part of the collection of Dobrudzha Agricultural Institute were used, as well the durum wheat parental forms (T. durum cv. Gergana and cv. Argonavt) and the wild species D. villosum.

The accession of D. villosum (2n = 2x = 14 (VV); family Poaceae, tribe Triticeae, subtribe Triticineae, genus Dasypyrum) was collected in Dobrich region in 2011.

Crosses Gergana × D. villosum and Argonavt × D. villosum were made conventionally, without embryo rescue in 2012; the obtained seeds (Gergana × D. villosum – 3 seeds and Argonavt × D. villosum – 8 seeds) were germinated and at tillering stage the plants (Gergana × D. villosum – 1 plant and Argonavt × D. villosum – 3 plants) were treated with colchicine in 2013. The seeds from the two obtained primary amphidiploids were multiplied several times

Anther culture. The experiment was carried out during 2016/2017. Anther donor plants were grown under greenhouse conditions. The seeds from the accessions were germinated in Petri dishes and then planted in plastic pots. Fifteen plants from each accession were grown in three pots, using 10 plants per genotype. Primary, seedling were vernalized at 4 °C (3000 lx, 16 h day/8 h night) for 45 days. After this period, the plants were transferred to a cold greenhouse (5–15 °C) for about three months, and the temperature was later increased to 15–20 (25) °C. Tillers bearing spikes containing anthers with microspores at mid- to late uninucleate stage were cut, put in a vessel with water and pretreated at 4 °C for 8–9 days. Ten spikes from each genotype were collected. Cold pretreated spikes were surface sterilized with 70 % ethanol under aseptic conditions. Sixty anthers from each spike were placed in test tubes with 20 ml P2 induction medium (Chuang et al., 1978). The anthers were cultured at 28 °C in darkness for about 60 days. After the 30th day, they were periodically checked for induction of embryogenic structures (calli and embryoids), which were transferred to test tubes with 10 ml regeneration medium (Zhuang, Jia, 1983) and cultured at 25 °C (3000 lx, 16 h day/8 h night). Green and albino regenerants were counted after 30 days.

The androgenic response was estimated by the following traits: callus induction (CI) (number of embryogenic structures induced per 100 cultured anthers, %), plant regeneration (PR) (number of regenerated green and albino plants per 100 embryogenic structures, %), frequency (yield) of green plants (YGR) (number of regenerated green plants per 100 cultured anthers, %) and frequency (yield) of albino plants (YAR) (number of regenerated albino plants per 100 cultured anthers, %).

Statistics. The obtained results were summarized over genotypes and parameters. One way ANOVA was carried out with the aim of determining the effect of the genotype on the studied parameters to estimate their androgenic response. Significant differences between the amphidiploids and their parental forms were calculated based on the Duncan test. To process the data, software MS Office Excel 2003 was used, and to perform ANOVA and the Duncan test – IBM SPSS Statistics v.19.

## Results

The results on the androgenic potential of the investigated amphidiploids and their parental forms (Table 1) showed that accession 1dv had the highest callus induction (30.7 %), and durum wheat Argonavt – the lowest (2.1 %). Between the parental forms, there were no significant differences (both between the durum wheat cultivars and between the species durum wheat and D. villosum). The two amphidiploids differed significantly by their callus induction, which was probably related to the effect of the maternal component.

**Table 1.**
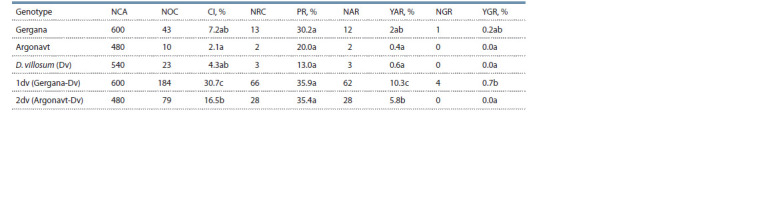
Androgenic response of parental forms and durum wheat-D. villosum amphidiploids Note. NCA – number of cultivated anthers; NOC – number of obtained calli; CI – callus induction; NRC – number of regenerative calli; PR – plant regeneration; NAR – number of albino regenerants; YAR – yield of albino regenerants; NGR – number of green regenerants; YGR – yield of green regenerants.

The yield of green plants, averaged for the entire investigated set, was extremely low. In the entire experiment, only 5 green regenerants were produced, one of them being from the durum wheat cultivar Gergana, and the other 4 – from the amphidiploid 1dv (Gergana-D. villosum). No green regenerants were obtained from cultivar Argonavt and from the amphidiploid 2dv. Also, no green plants were produced from the wild species D. villosum. Although there was a rather small number of the obtained plants for formulating a general tendency for the effect of the parental forms, the presence of green plants in cultivar Gergana and the amphidiploid, in which it was involved, was probably due to genotypic specificity.

The albino plants considerably exceeded the green regenerants. In practice, they were predominant with regard to the total number of regenerants. The amphidiploid 1dv again had the highest yield of albino plants (10.3 %), and the lowest values were observed in cultivar Argonavt (0.4 %). The tendency in yield of obtained albinos largely followed the tendency of callus induction. The two amphidiploids significantly differed from the parental forms by their values, as well as between themselves (10.3 and 5.8 %, respectively). Meanwhile, significant differences between the two durum wheat cultivars and between the durum wheat and the wild species were not registered. The higher yield values of the albino plants in the amphidiploid 1dv may be related to the higher responsiveness of cultivar Gergana, which was the maternal component of this amphidiploid, although the difference between Gergana and Argonavt was not significant.

On the whole, plant regeneration, expressed as a number of regenerants per 100 embryogenic structures, was comparatively low. The highest values were read in the two investigated aphidiploids (35.9 and 35.4 %, respectively), and the lowest – in the wild species D. villosum (13.0 %). This parameter did not follow the tendency observed in the values of callus induction and yield of green and albino regenerants. There were no significant differences between any of the studied accessions. However, higher plant regeneration was registered in the amphidiploids, in comparison to cultivar Argonavt and the wild species, and the difference with cultivar Gergana was considerably lower. The differences not being significant was an indication that the regeneration potential of all studied accessions was practically identical, and the differences formed were entirely random. The total number of regenerants, however, expressly followed the tendency of callus induction and yield of albino plants. The higher responsiveness to anther culture of the two investigated amphidiploids in comparison to either of the parental forms could be clearly observed in this parameter.

The results from the analysis of the variance of the studied parameters (Table 2) showed that the genotype had a significant effect on the parameters callus induction and yield of albino regenerants. This allows supposing that the separate accessions gave specific responses and that there are significant differences between them, as determined by the Duncan test that was carried out. At the same time, the effect of the separate accessions on the plant regeneration and the yield of green plants was not significant. Worth mentioning are accessions Gergana and Gergana-D. villosum, in which higher responsiveness to anther culture was observed, in general. Nevertheless, these results do not give a definite answer to the question of whether the amphidiploids are different as a biologically distinct organism from the two parental forms with regard to their androgenic response.

**Table 2.**
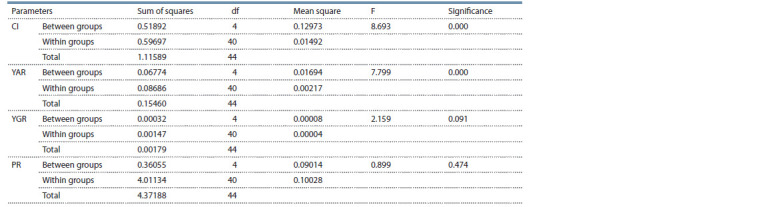
ANOVA according to factor “accession” of the studied accessions Note. CI – callus induction; YAR – yield of albino regenerants; YGR – yield of green regenerants; PR – plant regeneration.

When summarizing the results at the level of the species, a clear tendency of the amphidiploid T. durum-D. villosum having significantly higher callus induction and yield of albino regenerants was evident (Table 3).

**Table 3.**
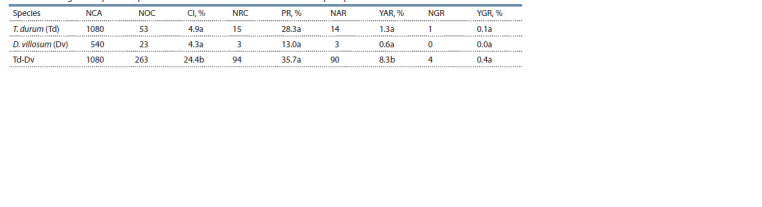
Androgenic response of parental forms and durum wheat-D. villosum amphidiploid Note. NCA – number of cultivated anthers; NOC – number of obtained calli; CI – callus induction; NRC – number of regenerative calli; PR – plant regeneration; NAR – number of albino regenerants; YAR – yield of albino regenerants; NGR – number of green regenerants; YGR – yield of green regenerants.

Simultaneously, significant differences between the two parental forms were not observed, the values of both parameters being significantly lower in them. The yield of green plants from the parental forms and from the amphidiploid was extremely low and did not allow forming a clear tendency. In this case, the production of green regenerants was rather random, without observing significant differences between the investigated species. Plant regeneration, at the levels of both genotype and species, did not differ as a tendency. The observed differences were not significant (see Tables 3 and 4), which indicated that the studied amphidiploid did not differ from the parental forms by its regeneration capacity.

**Table 4.**
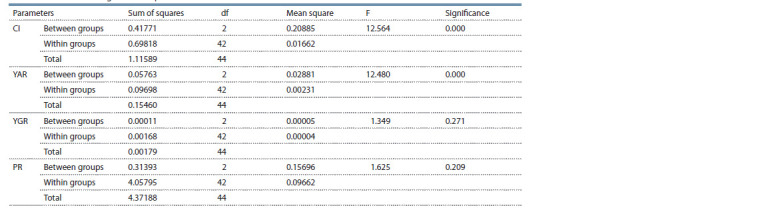
ANOVA according to factor “species” of the studied accessions Note. CI – callus induction; YAR – yield of albino regenerants; YGR – yield of green regenerants; PR – plant regeneration.

## Discussion

Concerning the results obtained on the androgenic response of the used accessions, it should be emphasized, that no source was found in world literature that would present data on the amphidiploid T. durum-D. villosum or the species D. villosum. An exception was the research of X. Chen et al. (1996), who suggested applying the anther culture method on hybrids (not amphidiploids) of the F1 (T. durum × D. villosum). These authors reported successful production of amphidiploids, regenerated from colchicine-treated calli. At the same time, there are researches on the use of tissue cultures on three component hybrids T. aestivum × (T. durum-D. villosum). H. Li et al. (2005) reported lines with high powdery mildew resistance obtained from such hybrids through the method of embryo rescue and subsequent anther culture.

D. Plamenov et al. (2009), when investigating the androgenic response of accessions from the amphidiploid T. durum- T. monococcum ssp. aegilopoides, came up with results different from ours. The reported callus induction was 3.3–11.7 % for the two studied accessions, the plant regeneration was considerably higher, 33.8–68.4 %, respectively, and the albino regenerants yield was 1.9–3.2 %. At the same time, the yield of green plants (0.4–0.8 %) was a little higher than the data we obtained in our experiment (0.0–0.7 %). These authors reported a total of seven regenerated plants from both accessions, this parameter being significant, unlike the results we obtained. Using anther culture in the amphidiploid Ae. variabilis-S. cereale, and P2 medium, A. Ponitka et al. (2002) observed 1.4–15.7 % of callus induction, and on C17 medium – 20.0–65.2 %. Subsequently, the authors reported 0.1–13.4 % yield of green regenerants using 190-2 regeneration medium. It was found out that the androgenic response was strongly dependent on the genotype, similar to the results of the experiment we conducted. Successful regeneration of green plants through the method of anther culture has also been reported for an aneupolyhaploid of Thynopyrum ponticum (Wang et al., 1991), for the amphidiploid Festuca pratensis- Lolium multif lorum (Lesniewska et al., 2001; Zwierzykowski et al., 2001; Rapacz et al., 2005) and the amphidiploid Cyclamen persicum-C. purpurascens (Ishizaka, 1998).

In contrast to these results, the parental forms were characterized with much lower androgenic response. This was confirmed by the absence of callus induction in Ae. variabilis and rye, reported by A. Ponitka et al. (2002), and also in the species T. monococcum ssp. aegilopoides in the research of D. Plamenov et al. (2009). Durum wheat is also characterized by weak androgenic response, in general. M. Doğramacı- Altuntepe et al. (2001), using 10 durum wheat genotypes, obtained only 248 green regenerants from 86,400 anthers (0.29 %). F. J’Aiti et al. (1999), investigating 15 durum wheat genotypes and 7500 cultivated anthers, obtained just three albino regenerants and one green plant.

L. Cistúe et al. (2006), on the other hand, reported significantly higher production of green plants, but including 6-benzylaminopurine or 6-furfurilaminopurine in the induction medium (C17). In more recent researches, the production of haploids, even by the method of isolated microspores, has been of extremely low efficiency in durum wheat (Slama-Ayed et al., 2019). These results entirely corresponded to the data we obtained with regard to the two cultivars Argonavt and Gergana. Clear genotypic specificity was observed in the better response of Gergana to anther culture as compared to Argonavt, although the difference was not statistically significant. It is probable that this tendency is the reason for the amphidiploid Gergana-D. villosum having better responsiveness to anther culture. In this respect, the amphidiploid T. durum-D. villosum we investigated, and the amphidiploids reported by A. Ponitka et al. (2002) and D. Plamenov et al. (2009) were closer by their androgenic response to the response of triticale (which is a typical amphidiploid crop) than to the response of the parental forms. J. Pauk et al. (2000), K. Marciniak et al. (2003), C. Lantos et al. (2014) and H. Stoyanov et al. (2019) demonstrated that in triticale the albino regenerants are often predominant, similar to the amphidiploid we studied. The values of the green regenerants in triticale also varied (from 0.9 to 27.9 %, but more often within 3–6 %), according to data from various researches (Gonzales, Jouve, 2000; Marciniak et al., 2003; Banaszak, 2011; Lantos et al., 2014).

In contrast to the above responses of the parental forms Ae. variabilis, S. cereale and T. monococcum ssp. aegilopoides, our study, although limited in volume, demonstrated the comparatively good responsiveness of the species D. villosum to anther culture. This is the first time when results on regenerants from this species (although only albinos) are being reported. At the same time, it should be emphasized that until this moment results from testing of the reaction of D. villosum to the anther culture method have never been reported. This is highly significant for the breeding of the wheat species since it would allow transferring genes from the wild species through the methods of wide hybridization and anther culture more easily, quickly and efficiently. X. Chen et al. (1996) and C. Li et al. (2000) reported common wheat lines resistant to powdery mildew, which were obtained by crossing common wheat to the amphidiploid T. durum-D. villosum, followed by embryo rescue and anther culture. Such results showed that the combination of wide hybridization with the method of anther culture is an efficient tool that can be used in the breeding of different cereal crops.

## Conclusion

Based on the presented results, the following conclusions could be made:

1. For the first time, results on the androgenic response (callus induction, plant regeneration, yield of albino plants, yield of green plants) of the amphidiploid T. durum-D. villosum and of the parental component D. villosum are being reported.

2. The callus induction of the two studied amphidiploids differed significantly from that of the parental forms (2.1–7.2 %), being considerably higher – 30.7 and 16.5 %, respectively.

3. The plant regeneration of the investigated accessions varied within a certain range (13.0–35.9 %), the differences not being statistically significant. This indicated that in spite of the differences in the callus induction, the amphidiploids did not practically differ from the parental forms by their regeneration capacity.

4. Although plant regeneration was observed in all studied accessions, the yield of albino plants considerably exceeded the yield of green regenerants and followed the tendency observed in callus induction – the two amphidiploids had significantly higher values. At the same time, green plants were registered only in the amphidiploid Gergana-D. villosum and in the parental form durum wheat Gergana. Such results emphasized the genotypic specificity of the response to anther culture

5. Plants were regenerated from the species D. villosum, although only albinos, which indicated its good responsiveness to anther culture. This, together with the good response of the amphidiploids with the participation of this species, makes their practical use, in combination with the anther culture method, highly valuable for improving the cereals.

## Conflict of interest

The authors declare no conflict of interest.
